# Objective assessment of tumour response to therapy based on tumour growth kinetics

**DOI:** 10.1038/bjc.2011.436

**Published:** 2011-10-25

**Authors:** E Mehrara, E Forssell-Aronsson, P Bernhardt

**Correction to**: *British Journal of Cancer* (2011) **105**, 682–686; doi:10.1038/bjc.2011.276

During the final editing of the above paper before its publication, errors were, unfortunately, introduced in equations 5 and 7. In both the equations, a minus sign had been deleted. The correct equations are now shown below.

Equation 5: 
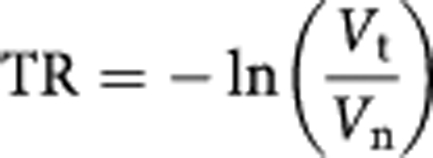


Equation 7: 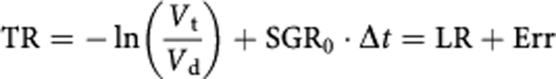


The authors and publishers apologise for any inconvenience this may have caused.

